# Improving Surgical Safety Through Checklist Compliance: Lessons From a Clinical Audit in a Teaching Hospital

**DOI:** 10.7759/cureus.99701

**Published:** 2025-12-20

**Authors:** Sarah Mohammed, Hebah Abobakr, Mohammed Abdullah, Badr Baras, Mazin Alzebeer, Taha Zaman, Mohamed A Eldaw, Muhammed Raheel, Israa Alamin

**Affiliations:** 1 Surgery, Omdurman Military Hospital, Omdurman, SDN; 2 Medicine and Surgery, Al Sheriani Medical Center, Khartoum, SDN; 3 Cardiology, Prince Sultan Cardiac Center (PSCC), Abha, SAU; 4 Surgery, Dr Sulaiman Al-Habib Hospital, Jeddah, SAU; 5 Faculty of Health and Life Sciences, Coventry University, Coventry, GBR; 6 Surgery, Bahawal Victoria Hospital, Bahawalpur, PAK; 7 Medical Education and Simulation, University of Gadarif, Gadarif, SDN

**Keywords:** clinical audit, compliance, educational intervention, omdurman teaching hospital, patient safety, sudan, surgical safety checklist

## Abstract

Background

The World Health Organization (WHO) Surgical Safety Checklist (SSC) is a validated tool to improve perioperative safety and reduce preventable complications. Despite being introduced worldwide, compliance with the SSC remains inconsistent, especially in limited-resource settings. This study aimed to assess compliance with the SSC, identify barriers to its implementation, and evaluate the impact of an educational intervention at Omdurman Teaching Hospital, Omdurman, Sudan.

Methods

An observational clinical audit was conducted in the Department of Surgery at Omdurman Teaching Hospital between March and April 2025. A total of 100 operations were observed: 50 during the first audit cycle (baseline) and 50 during the second cycle (post-intervention). Data were collected using a structured tool adapted from the WHO SSC, recording whether checklist items were performed and/or verbalized during the Sign-In, Time-Out, and Sign-Out phases. An educational intervention, consisting of departmental seminars, posters, and reminders, was introduced between the two cycles. Staff feedback regarding barriers to SSC compliance was also obtained. Data were analyzed using IBM SPSS Statistics for Windows, Version 26 (Released 2018; IBM Corp., Armonk, NY, USA), with results expressed as frequencies and percentages.

Results

Baseline compliance with the SSC was suboptimal. Sign-In, Time-Out, and Sign-Out were performed in 60%, 52%, and 30% of cases, respectively, during Cycle 1. Following the intervention, compliance improved significantly: Sign-In to 86%, Time-Out to 80%, and Sign-Out to 88%. Documentation improved from 34% to 70%, while patient board completion rose from 28% to 64%. Verbalization of checklist items remained weak (e.g., Sign-Out read aloud: 0% vs. 12%). Staff cited lack of awareness (90%), absence of responsibility (75%), and perceptions of increased workload (60%) as key barriers.

Conclusion

Implementation of the WHO SSC at Omdurman Teaching Hospital demonstrated measurable improvements in compliance after a simple educational intervention. However, persistent gaps in verbalization and staff perceptions highlight the need for ongoing training, leadership engagement, and cultural change to sustain long-term adherence and strengthen surgical safety in Sudan.

## Introduction

Surgical care is a critical component of healthcare systems, with an estimated 313 million procedures performed worldwide each year [1]. Despite advances in surgical techniques and anesthesia, surgery continues to carry significant risks, and adverse events remain a leading cause of morbidity and mortality [2]. Globally, an estimated 313 million surgical procedures are performed annually, and adverse perioperative events remain a major source of preventable morbidity and mortality, underscoring the importance of system-level safety interventions such as the World Health Organization (WHO) Surgical Safety Checklist (SSC) [1,3]. Importantly, many of these events are preventable through structured safety practices.

To enhance perioperative safety, the WHO launched the “Safe Surgery Saves Lives” initiative in 2008, introducing the SSC as a standardized tool to minimize avoidable errors [3]. The SSC consists of three phases: Sign-In (before induction of anesthesia), Time-Out (before skin incision), and Sign-Out (before the patient leaves the operating theatre). Each SSC phase is designed to reinforce team communication and shared situational awareness: Sign-In verifies patient identity and anesthesia readiness, Time-Out allows all team members to confirm the planned procedure and critical steps, and Sign-Out ensures counts and postoperative plans are clear - interventions that have been associated with reductions in communication-related errors and adverse events. Each phase is designed to reinforce communication, teamwork, and systematic verification of critical safety steps [4].

Evidence from multiple countries has shown that SSC implementation reduces postoperative complications and mortality while improving teamwork and patient safety culture [5,6]. For instance, a landmark study demonstrated a reduction in major complications from 11% to 7%, and mortality from 1.5% to 0.8% after checklist adoption [4]. However, compliance with the checklist remains inconsistent across institutions. Reports from low- and middle-income countries (LMICs) indicate compliance rates as low as 39.7% in Ethiopia [7], and highlight barriers such as limited training, staff shortages, and perceptions of increased workload [8,9].

In Sudan, where healthcare facilities often face resource constraints, high surgical loads, and inadequate staffing, ensuring surgical safety is particularly challenging. Despite the global evidence supporting the SSC, little is known about its adoption and compliance in Sudanese hospitals. This study was therefore conducted at Omdurman Teaching Hospital to evaluate compliance with the WHO SSC, identify barriers to its use, and propose strategies to improve its implementation in the Sudanese context.

We hypothesized that the educational intervention would significantly increase compliance with the WHO SSC in measured checklist domains, particularly documentation and Sign-Out verbalization, between Cycle 1 and Cycle 2.

## Materials and methods

**Figure 1:**
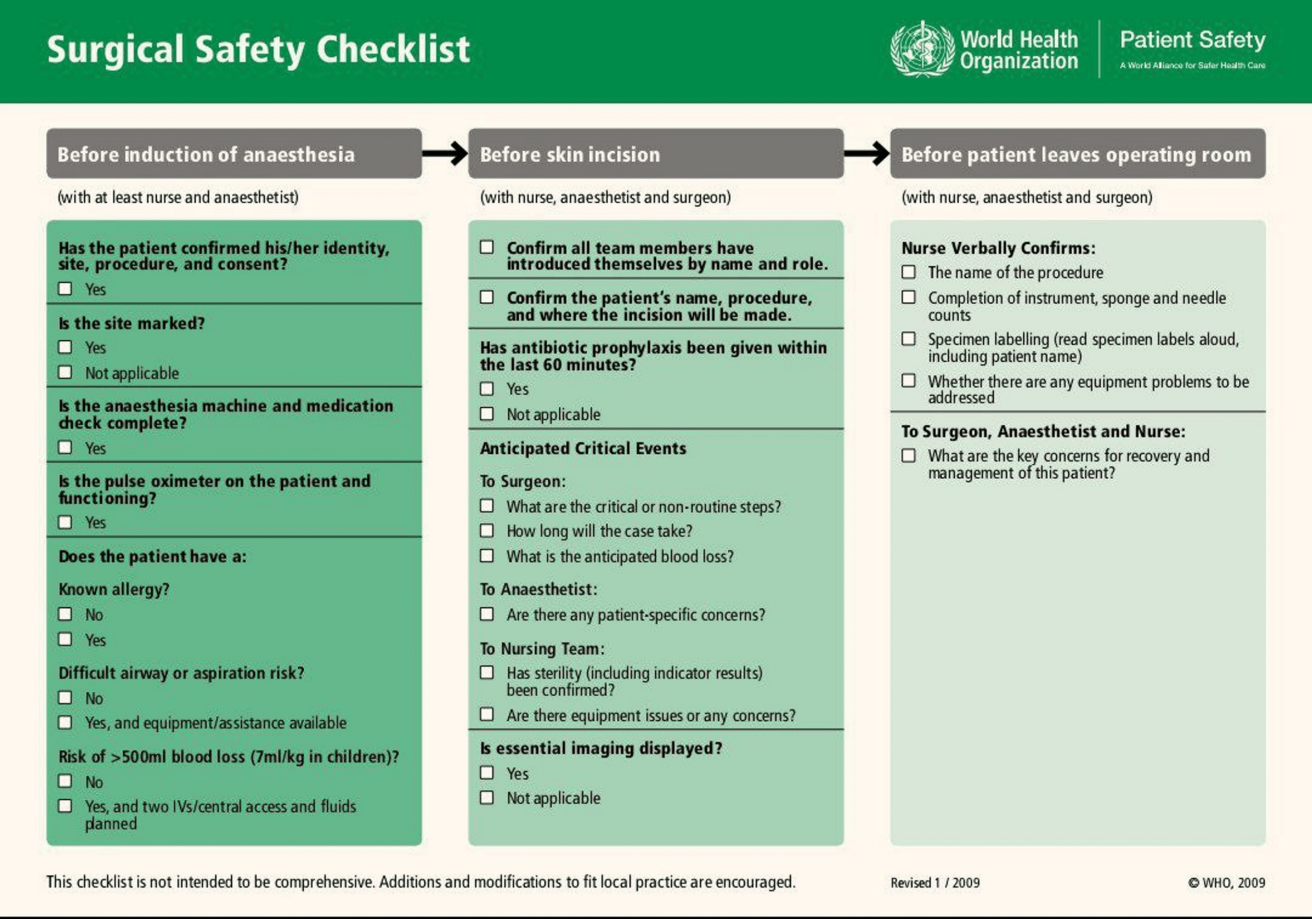
WHO surgical safety checklist Image credit: [10]

All surgical procedures performed in the main operating theaters during the study period were eligible for inclusion, encompassing both emergency and elective operations. Patients undergoing minor procedures outside the main theatres, such as in dressing rooms or minor surgery units, were excluded. Data were collected using a structured observation checklist adapted from the 2009 version of the WHO SSC. This tool is free to use and open-access, provided by the WHO [10]. The tool captured demographic characteristics (age, sex, and type of surgery), compliance with each of the three critical SSC phases (Sign-In, Time-Out, and Sign-Out), as shown in Figure 1, and documentation in patient files and theater boards. In addition, key performance-related items were assessed, including surgical site marking, confirmation of anesthesia machine and medication checks, availability of a pulse oximeter, display of essential imaging, sponge/needle/instrument counts, and specimen labeling.

This observational clinical audit was conducted at the Department of Surgery, Omdurman Teaching Hospital, Omdurman, Sudan, one of the largest tertiary care centers in the country. The study followed the Standards for Quality Improvement Reporting Excellence (SQUIRE 2.0) guidelines, which are freely available as an open-access resource for researchers [11], and was carried out over a two-month period, from March to April 2025. Ethical approval was obtained from the Hospital Research and Ethics Committee of Omdurman Teaching Hospital, and patient confidentiality and anonymity were strictly maintained. No identifiable patient data were collected.

The audit was conducted in two cycles. In Cycle 1 (baseline), compliance with the SSC was observed without staff awareness, to minimize observer bias. Between the two cycles, a one-week educational intervention was implemented, consisting of a departmental seminar on the importance of the SSC, circulation of printed reminders and posters within the operating theatres, and direct instructions from the Head of Department encouraging adherence. In Cycle 2 (post-intervention), compliance was reassessed using the same methodology and observation tool.

Of the 100 observed procedures, 62% were emergency surgeries and 38% were elective procedures. The distribution was comparable between cycles: Cycle 1 included 31 emergency and 19 elective procedures (n = 50), and Cycle 2 included 31 emergency and 19 elective procedures (n = 50). This ensured that any changes in checklist compliance were unlikely to be explained by differences in case mix between cycles.

At the conclusion of Cycle 2, qualitative feedback was collected from operating theatre staff, including surgeons, anesthetists, nurses, and technicians, through structured interviews. Staff were asked to identify perceived barriers to SSC compliance across four domains: awareness and training, feasibility, motivation, and perceived necessity.

During observations, the core operating team was defined as the surgeon, the anesthetist, and the circulating nurse. The presence of all three core team members was recorded for each observed case. Full core-team presence (surgeon + anesthetist + circulating nurse) was observed in 92% (92/100) of procedures; at least a surgeon and an anesthetist were present in 100% of procedures.

Data were entered and analyzed using IBM SPSS Statistics for Windows, Version 26 (Released 2018; IBM Corp., Armonk, NY, USA). Quantitative variables, such as patient age, were summarized as mean ± standard deviation (SD), whereas categorical variables, including sex and checklist compliance, were presented as frequencies and percentages. Comparisons of compliance between Cycle 1 and Cycle 2 were performed using the Chi-square or Fisher’s exact test, with p < 0.05 considered statistically significant.

Statistical comparisons were therefore performed using the percentages reported in the manuscript and reconstructed cell counts (n = 50 per cycle), derived from those percentages. This approach reported audit findings, and p-values should therefore be interpreted in that context.

## Results

A total of 100 surgical procedures were observed: 50 during the first audit cycle (Cycle 1) and 50 during the second audit cycle (Cycle 2). The mean age of patients was 39.8 ± 17.5 years, with 54% males and 46% females. Of the operations, 62% were emergency cases and 38% were elective procedures.

Compliance before and after intervention

Compliance with the WHO SSC was initially low during Cycle 1 but demonstrated marked improvement in Cycle 2 following the educational intervention. Patient board completion increased from 28% to 64%, while documentation of the checklist in the patient file improved from 34% to 70%. The Sign-In process was performed in 60% of cases in Cycle 1 and rose to 86% in Cycle 2, whereas Time-Out compliance improved from 52% to 80%. Sign-Out showed the most substantial improvement, increasing from 30% to 88%. Despite these gains, the verbalization of checklist steps remained the weakest area, although modest progress was observed, with Sign-Out verbalization improving from 0% to 12%, as shown in Table 1.

**Table 1:** Marked improvement in compliance with the WHO Surgical Safety Checklist after the intervention, especially in the Sign-Out phase (30% → 88%). However, verbalization steps remained low despite modest gains. WHO Surgical Safety Checklist [10].

Cycle 1 (n = 50)	Cycle 2 (n = 50)	Change
28%	64%	36%+
34%	70%	36%+
60%	86%	26%+
10%	18%	8%+
52%	80%	28%+
6%	14%	8%+
30%	88%	58%+
0%	12%	12%+

We reconstructed counts from the percentages reported in Table 1 (n = 50 per cycle) and performed statistical tests. Counts (Cycle 1/Cycle 2) and p-values are as follows: (i) Patient board completion: 14/50 → 32/50; χ² p = 0.00065 (Fisher p = 0.00057), significant. (ii) Documentation completion (checklist in patient file): 17/50 → 35/50; χ² p = 0.00067 (Fisher p = 0.00059), significant. (iii) Sign-In performed: 30/50 → 43/50; χ² p = 0.0069 (Fisher p = 0.0063), significant. (iv) Item with change from 10% to 18%: 5/50 → 9/50; χ² p = 0.36 (Fisher p = 0.34), not statistically significant. (v) Time-Out performed: 26/50 → 40/50; χ² p = 0.0026 (Fisher p = 0.0025), significant. (vi) Item with change from 6% to 14%: 3/50 → 7/50; χ² p = 0.317 (Fisher p = 0.318), not statistically significant. (vii) Sign-Out performed: 15/50 → 44/50; χ² p < 0.001 (χ² p = 1.25 × 10⁻⁸; Fisher p ≈ 3.76 × 10⁻⁹), highly significant. (viii) Verbalization step (0% → 12%): 0/50 → 6/50; χ² p = 0.035 (Fisher p = 0.027), significant. Where small expected cell counts (<5) occurred, Fisher’s exact test was reported alongside chi-square results. These p-values have been inserted into the Results section (paragraph describing compliance changes).

Staff feedback

At the end of Cycle 2, structured interviews were conducted with 20 staff members, including surgeons, anesthetists, nurses, and theatre technicians, to explore barriers to SSC compliance. The most frequently reported barrier was lack of awareness or training, identified by 90% of participants. Additionally, 75% highlighted the absence of clear responsibility for initiating the checklist, while 60% perceived the SSC as time-consuming. Furthermore, 45% of respondents believed that many steps within the checklist were unnecessary or redundant.

Core team attendance was high: all three core members (surgeon, anesthetist, and circulating nurse) were present in 92% of observed procedures (46/50 in Cycle 1; 46/50 in Cycle 2), ensuring that changes in checklist use were not attributable to asymmetric team availability.

## Discussion

This clinical audit evaluated compliance with the WHO SSC at Omdurman Teaching Hospital and demonstrated that, although baseline adherence was suboptimal, significant improvements were observed following a simple educational intervention. The most notable improvement was seen in the Sign-Out phase, where compliance rose from 30% to 88%. This finding is consistent with previous studies in Pakistan [12] and Nigeria [13], which reported that targeted training and departmental support can substantially improve adherence to checklist items, particularly those related to teamwork and communication.

However, despite the overall improvement, verbal components of the checklist (read-aloud steps) remained poorly implemented. For example, introducing team members during the Time-Out and verbalizing the Sign-Out were among the least performed actions. Similar trends have been documented in Ethiopia [7] and Sweden [14], where surgical staff considered verbal communication unnecessary or time-consuming. This suggests that, beyond awareness, there are cultural and behavioral barriers that hinder full SSC adoption, particularly in LMICs.

We acknowledge that barriers described in high-income settings, like Sweden, often reflect different underlying drivers of workflow design and entrenched professional norms compared to typical LMIC challenges (resource constraints, staffing shortages, and limited training). Thus, while some behavioral barriers overlap across settings, interventions must be locally adapted - for example, focusing on low-cost system supports and leadership-driven accountability in resource-limited hospitals.

Another important observation was that compliance with documentation and patient board completion improved but did not reach 100%. This highlights the need for institutional enforcement and integration of the SSC into standard operating protocols. Previous studies emphasize that the sustainability of SSC use requires ongoing monitoring, leadership involvement, and staff accountability [15,16].

Feedback from theater staff in our audit revealed common barriers: lack of training, absence of a designated initiator, and the perception that the SSC prolongs operating time. These barriers echo findings from studies in England [8] and France [9], where resistance was largely due to workload concerns and skepticism about the checklist’s benefits. Addressing these barriers requires not only training but also leadership-driven cultural change, emphasizing that patient safety is enhanced rather than delayed by checklist use.

Our findings support the growing body of evidence that educational interventions are effective in improving SSC compliance, but they also indicate that long-term sustainability will depend on embedding the SSC into hospital policy, regular audits, and possibly local adaptations to fit the Sudanese surgical context.

## Conclusions

This audit demonstrated that implementation of the WHO SSC at Omdurman Teaching Hospital led to significant improvements in compliance following a simple educational intervention. The greatest gains were seen in documentation and the Sign-Out phase, reflecting improved attention to patient safety practices. However, verbal communication components of the checklist remained poorly adopted, highlighting the need for continuous training, strong leadership support, and cultural change to ensure sustained compliance. Integrating the SSC into routine surgical protocols can play a vital role in enhancing perioperative safety and improving surgical outcomes in Sudan.

Recommended institutional mechanisms to support sustainability include incorporating the SSC into mandatory perioperative documentation, with required sign-off fields in patient charts and theatre boards; designation of a “checklist initiator” (rotated among staff) with explicit responsibility; quarterly departmental audits, with feedback to teams; brief, task-focused refresher training during departmental meetings; and leadership endorsement (Head of Department), with inclusion of checklist adherence in departmental performance metrics.
